# Epidemiology of High-Risk Sleep Phenotypes in a Diverse Cohort of Older Adults

**DOI:** 10.1093/geroni/igaf122.1914

**Published:** 2025-12-31

**Authors:** Brienne Miner, Jarett Talarczyk, Clemence Cavailles, Carrie Peltz, Kristine Yaffe

**Affiliations:** Yale University, New Haven, Connecticut, United States; Yale University, New Haven, Connecticut, United States; University of California, San Francisco, San Francisco, California, United States; Northern California Institute for Research and Education, San Francisco, California, United States; University of California, San Francisco, San Francisco, California, United States

## Abstract

**Background:**

Insomnia with objective short sleep duration (ISSD) and objective long sleep duration (LS) are high-risk sleep phenotypes that have not been examined in a diverse group of older adults.

**Methods:**

We studied 1001 adults in the DORMIR study (average age 64.4[8.6] years, 65.7% female; 28.4% Black, 31.6% Hispanic, 40.1% non-Hispanic White). Insomnia was defined as self-reporting any of the following > =3 times/week: trouble getting to sleep within 30min, waking in the middle of the night/early morning or taking sleep medication. Objective overnight sleep duration was assessed by actigraphy (averaged over 5.9[0.9] days). We examined ISSD (insomnia and sleep duration< 6h), LS (sleep duration>8h), and normal sleep (NS; no insomnia and sleep duration=6-8h). Using ANOVA F-tests or chi-square, we assessed phenotype prevalence overall and according to race/ethnicity, and also examined, within each race/ethnicity group, whether comorbidities (e.g., depression, anxiety, dementia status, physical impairment [Short Physical Performance Battery< 10]) differed among phenotypes.

**Results:**

ISSD prevalence was 8.1% overall and was higher among Blacks (13.4%; p < 0.001). LS prevalence was 22.1% overall and was higher among Whites (27.2%; p < 0.001). Compared to NS, depression was higher in ISSD and LS across all groups. Anxiety was higher in Hispanics and Whites with ISSD and LS compared with NS. Across all groups, physical impairment was significantly higher in LS compared with NS. Dementia status was not different across phenotypes.

**Conclusion:**

The prevalence of high-risk sleep phenotypes differs across race/ethnicity groups, while comorbidities associated with phenotypes were similar. Future work should examine the association of phenotypes with adverse outcomes.

